# Root-Derived Proteases as a Plant Tool to Access Soil Organic Nitrogen; Current Stage of Knowledge and Controversies

**DOI:** 10.3390/plants10040731

**Published:** 2021-04-08

**Authors:** Bartosz Adamczyk

**Affiliations:** The Natural Resources Institute, Luonnonvarakeskus, Viikinkaari 4, 00790 Helsinki, Finland; bartosz.adamczyk@luke.fi; Tel.: +358-295322222

**Keywords:** nitrogen, proteolysis, soil

## Abstract

Anthropogenic deterioration of the global nitrogen (N) cycle emerges mainly from overuse of inorganic N fertilizers in nutrient-limited cropping systems. To counteract a further dysregulation of the N cycle, we need to improve plant nitrogen use efficiency. This aim may be reached via unravelling all plant mechanisms to access soil N, with special attention to the dominating high-molecular-mass N pool. Traditionally, we believe that inorganic N is the only plant-available N pool, however, more recent studies point to acquisition of organic N compounds, i.e., amino acids, short peptides, and proteins. The least known mechanism of plants to increase the N uptake is a direct increase of soil proteolysis via root-derived proteases. This paper provides a review of the knowledge about root-derived proteases and also controversies behind this phenomenon.

## 1. Introduction

Nitrogen (N) plays a central role in all living systems, acting as an ingredient of proteins, including enzymes and nucleic acids. Nitrogen limitation of cropping system entails the need to use N fertilizers to satisfy the need for high plant yield. However, overuse of synthetic fertilizers has deteriorated the biogeochemical N cycle [1] bringing the following environmental consequences: increased N_2_O (nitrous oxide) emissions, N deposition and eutrophication of water reservoirs. These repercussions are an outcome of the inability of agricultural ecosystems to assimilate or immobilize all the added N fertilizer, as only 30% to 50% of the applied inorganic N (IN) fertilizer is taken up by the plants and the rest is lost by run-off, leaching, or volatilization [2]. One of the solutions to decrease further anthropogenic deterioration of the N cycle is to improve the plant N use efficiency in fertilization [3,4]. To satisfy this aim, we need a better understanding of the N cycling including a holistic view of all plant strategies to access soil N pools. Traditionally, it is assumed that only the IN pool is available to plants and thus N fertilizer recommendations are based on a soil N-test or models that only take into account the IN sources. However, more recent work underlines the ability of plants to access organic N compounds, i.e., amino acids [5], short peptides [6,7] proteins, whole intact microorganisms [8,9,10,11]. In addition, it was proposed that plants affect soil proteolysis through root-derived proteases [9,12]. However, studies aiming to apprehend an active increase of soil proteolysis via secretion of enzymes provided controversial results and interpretations [13,14,15], as we still lack a mechanistic understanding of the phenomena. Though we know that endogenic proteases play a crucial role in numerous plant processes including development, defense, reproduction, embryogenesis, programmed cell death [16], and the MEROPS online database divides plant proteases into seven classes: serine, cysteine, aspartic, asparagine, threonine, glutamate, and metalloproteases [17], root-derived proteases are far less known. This review aims to disentangle some of the uncertainties behind the plant use of high-molecular-mass N compounds via root-derived proteases.

## 2. Nitrogen Cycle and Soil Proteolysis

**Figure 1:**
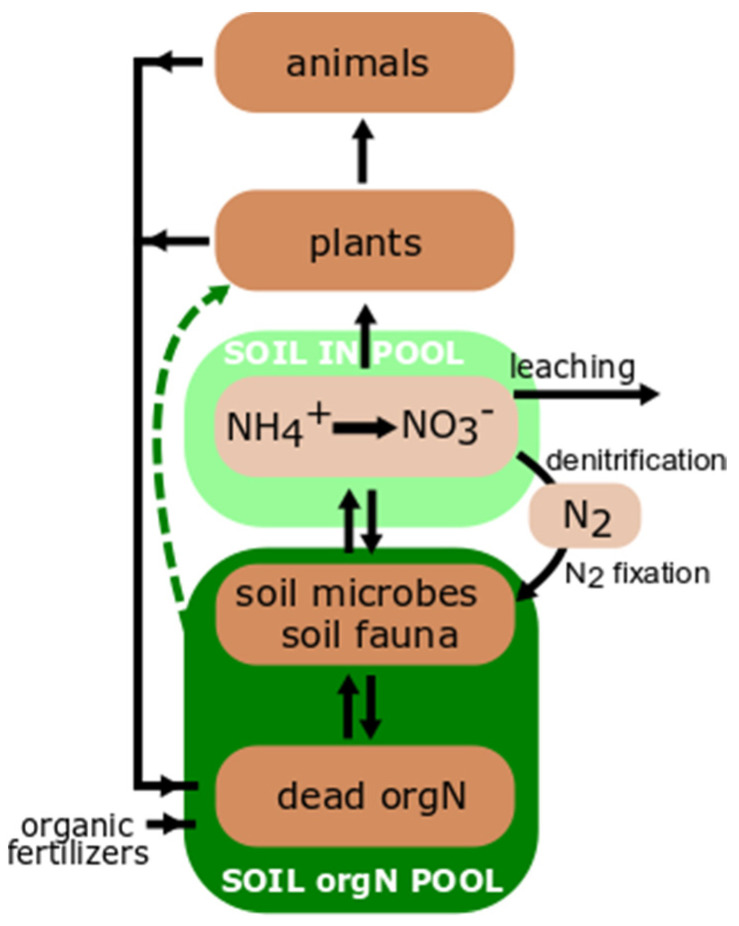
Nitrogen (N) cycle: pools and fluxes. Potential use of organic N pool marked in dashed green line. IN—inorganic N, orgN—organic N.

The nitrogen cycle and underlying processes have been studied for over a century [18], thus the main elements are known, though in-depth knowledge, especially about the role and accessibility of organic N pools for plants is still needed. The nitrogen pool is relatively stable, with the dominating reservoir in the air as N_2_, which is not plant-accessible. However, some Procaryota can reduce N_2_ via the nitrogenase complex, providing N to host plants (symbiotic N-fixing bacteria) or to the soil (free-living N-fixing bacteria). A pool of IN in the soil is mainly formed via ammonification, in which organic N compounds are decomposed to ammonia, further nitrified to nitrite and later nitrate, which may be denitrified by bacteria back to atmospheric N_2_ [19,20] (Figure 1, pools in light green and brown). Synthetic fertilizers like the commonly used ammonium nitrate (NH_4_NO_3_), significantly increase the IN pool in soil. However, soil N is mainly present in organic forms. Almost half of the soil N is in the form of proteins/peptides, and only less than one-fifth as IN [21,22]. The input of organic N is continuously provided via plant, animal, and microbial litter. In addition, organic fertilizers and ingredients of compost increase the soil organic N pool (Figure 1, pools in dark green and brown). Thus, fluxes of the organic N pool may have a crucial role for N cycling of ecosystems. Schimel and Bennett (2004) proposed that it is the rate at which proteinaceous compounds are depolymerized constitutes the rate-limiting factor for N mineralization. Depolymerization of proteins provides lower molecular mass organic N, which could be directly taken up by plants as peptides [7] and amino acids [5] or exposed to further decomposition to IN. The ability to take up directly organic N forms should be especially significant for organic farming compared to conventional farming, though the importance of organic N as a source of crop N under organic farming management systems is still poorly understood [23,24]. Considering the intensive competition between microorganisms and plants for N, the presence of effective mechanisms increasing the competitive abilities of plants to access organic N would explain the prevalence of plant over microbial biomass; globally, the plant biomass C exceeds these of microorganisms more than five times [25]. This dominancy cannot be simply explained by the ability of plants to assimilate C via photosynthesis, as N is one of the crucial nutrients necessary for growth, i.e., building biomass. Thus, plants need to compete and/or collaborate very effectively with microorganisms for N to obtain such a biomass dominance. Plants may enhance soil proteolysis indirectly via supporting rhizosphere microorganisms with easy-available C sources which may increase the decomposition of organic N (“rhizosphere priming effect”) [26], or directly via root-derived proteases [12]. Priming will not be discussed in this paper and explicit reviews may be found elsewhere (e.g., [27,28]). This review paper concentrates on root-derived proteases.

## 3. Soil Proteases and Root-Derived Enzymes

It is usually assumed that soil proteases originate from free-living and microbes associated with roots. Most of vascular plants live in symbioses with arbuscular mycorrhizal fungi (AMF), which increase the plant N pool via direct uptake of IN, amino acids, and increase of the volume of accessed soil [29,30]. However, AMF fungi rather do not secrete proteases like ectomycorrhizal fungi [31,32]. Therefore, the potential ability of agricultural plant roots to enhance proteolysis via root-derived proteases would be an important advantage to increase the organic N depolymerization and further the N uptake with help of AMF.

Roots are often neglected as a source of soil enzymes. However, a literature search may provide a surprising result; more than 100 proteins [33,34], including numerous enzymes, were ascribed as potentially root-secreted or associated with the root surface; Table 1 provides 10 examples of root-derived enzymes with their potential functions. As root-derived enzymes crucial for soil decompositionI am taking into account enzymes associated with the root surface [13,35], enzymes secreted via roots [12], or root border cells (RBCs). Root border cells are defined as cells which are alive after disconnecting with the root for a long time and they may even proliferate [36], acting supposedly in defense against abiotic and biotic stressors [36,37,38,39]. RBCs secrete numerous compounds, including cysteine protease [34,40]. Though root-surface associated enzymes are not exuded by roots, their importance can be even higher for soil proteolysis than exuded ones due to the spatial factor; enhanced proteolysis in the vicinity of root may decrease competition with root-associated microorganisms for proteolysis products, giving priority to the plant N uptake.

## 4. How Nitrogen Forms Affect Root Proteases—May Acid Phosphatases and P-Deficiency Give Some Hints?

On the contrary to root-derived proteases, root secretion of acid phosphatases is a better-known phenomenon. Thus, we could extrapolate mechanisms of acid phosphate secretion to root-derived proteases as the fate of soil phosphorus (P) and N share some similarities. As for N, P is also often mainly present in the soil in organic form, which is not available to plants [51]. A pool of inorganic P (Pi), thus a plant-available pool, is build up via hydrolysis of the ester bound C-O-P in organic P compounds (OP) catalyzed via acid phosphatases, which are released both by microorganisms [30,52] and by plant roots of numerous species (Table 1). For both of these enzymes, the existence of organic forms in the culture media increased their activities: root-derived acid phosphatases increased the activity from five to 11 times compared to culture media with Pi only [43,45], similarly, wheat seedlings increased the activity of root-derived proteases in the presence of protein [53,54]. 

Similarly to plants growing on a culture medium with protein as a sole N source, also plants cultivated on organic P as a sole P source showed decelerated growth compared to those growing on inorganic N or P only [15,55]. Moreover, the highest growth was observed for plants supplied with both OP and Pi [43], and for plants growing on media with both, IN and organic N in form of protein [9,56]. The best growth on organic and inorganic forms of N is in line with natural conditions, in which soils contain a mixture of IN and organic N [57] and a mixture of OP and Pi [51].

However, positive effects of protein on root-derived protease and plant growth were noted only for lower doses of proteins and higher concentrations of proteins decelerated root growth [14,58,59]. The growth inhibition could be caused by an increase of osmotic pressure due to addition of a high amount of proteins. High osmotic pressure decreases water absorption, inhibiting root growth [60]. In addition, proteins in high concentrations bind minerals affecting negatively plant growth [61].

There are also some differences between the activities of root-secreted proteases and acid phosphatases. Enhanced synthesis and excretion of acid phosphatase were documented under P-deficient conditions for several plant species [41,42,62,63,64]; on the other hand, activities of root-derived proteases decrease under N-deficient conditions, with no protein in the culture medium [53,56]. This difference may emerge from the nutrient economy; the investment of N into proteases is economically not favorable if no protein (i.e., substrate) is present in the culture medium. On the other hand, experiments with plants growing on P-deficient conditions rather do not include N-deficiency, so in these circumstances, plants may invest energy and N into phosphatases without loss of P to increase the P availability. The mechanism of decreased enzyme production in N-deficient conditions was shown for *Arabidopsis thaliana* seedlings investing less into defense-related peroxidases and chitinases under limited N supply although exposed to pathogens [65].

All in all, high similarities between the effect of P and N supply and root-derived acid phosphatases and proteases may help to disentangle the potential role in N cycling of the latter. 

## 5. Controversies about Root-Derived Proteases

Studies aiming to understand mechanisms behind plant access to soil proteins have provided controversial results and interpretations. This review paper revises the reasons behind such contradictions divided into different groups: experimental design-specific, plant physiology-specific, and methodological-specific ones.

### 5.1. Experimental Design Aspect

As mentioned above for proteases and acid phosphatases, specific conditions of growth may affect the activity of root-derived enzymes. In some experiments aiming to measure root-derived proteases only N-deficient conditions were used [12,13,35], in others also different forms of N were added to the culture medium [53,54]. We cannot expect that different experimental designs could provide the same outcome, thus different results origin from various N forms added to the medium. The plant N-deficiency could be enlarged by isolation of embryos from grains (e.g., wheat), providing a high N-deficiency, forcing the seedlings to search for N in order to survive [53]. Experimental designs with isolated embryos cannot be compared with those with no embryo isolation, especially for seedlings cultivated for a short time, where non-isolated embryos are still supported by N from storage materials (compare [14,53]). To overcome the problems mentioned above, we may design experiments with more treatments, reflecting different conditions also used in other papers, to make comparisons possible. Moreover, it would be beneficial to consider protein (i.e., substrate) in the culture medium if the aim is to study root-derived proteases (compare [9,53,58,59] vs. [13]). Strong conclusions with no consideration of differences in the experimental design, like the involvement of the protein in the culture medium for studies of root-derived proteases, should not be made.

### 5.2. Plant Physiology-Specific

Plant species differ significantly in N use efficiency, growth rate, amount of storage materials, and preferences to different N sources. For example, *Arabidopsis thaliana* and perennial *Lobelia anceps* differ in their preferences to use short peptides, amino acids, and inorganic N [6]. In addition, other mechanisms to access the high molecular weight organic N may differ between the species. As plant species differ in their preferences to N and P forms, analogically their strategies of N and P acquisition also differ [54,66,67,68]. 

In addition, also the age of seedlings may affect the use of different mechanisms to access the high molecular mass organic N. In line with that, we cannot expect similar behavior of very young seedlings and the older ones; for example, Chang and Bandurski (1964) revealed only negligible activity of root-secreted proteases from very young corn seedlings, however, such an exudation of proteases was observed for older seedlings of the same species [12]. The rationale behind the higher proteolytic activity of older seedlings may include a higher root-surface and demand for N. 

### 5.3. Methodological Aspects

In addition to the experimental design, conditions to grow the seedlings, species, age, and composition of the culture medium, we should consider also differences in the methodology used. The best examples here are studies of root-derived proteases. For instance, in [13] seedlings were growing in 50 mL containers with the culture medium which was replaced with a new medium after 7 days and this medium did not contain any proteins; later this medium was sampled with no further extraction and/or purification to enzyme activity measurements. The conclusion was that roots rather do not exude proteases [13]. On the contrary, in Adamczyk et al. (2008) root-secreted proteases were purified from the culture medium before activity measurements, and in [69] root-secreted proteases were also purified and concentrated with an absorption concentrator with a pore size of 7500 MWCO (Sartorius Stedim Biotech, Aubagne, France) before estimation of enzymatic activity. The preparation of the enzyme has a crucial effect on the results, and various methods to prepare the sample cannot provide very comparable results.

Another issue in the comparison of the root-derived protease activity is the use of a wide range of substrates. For example, the authors of [13] used fluorogenic substrate for leucine aminopeptidase to estimate root-derived proteases and compared their results with other studies where a different substrate [53], or methodology [9] were used. Aminopeptidases are exopeptidases removing amino acids from the amino-terminal end, and they release usually one amino acid residue. However, other studies showed that proteases exuded by roots are rather endopeptidases, acting on the carboxy terminus of the protein chain, thus releasing short peptides but not individual amino acids [70], which could be in line with plant preferences to short peptides over single amino acids [6]. In addition, carboxypeptidase secreted by *Allium porrum* roots showed similarities to cysteine protease from *Arabidopsis thaliana* [70], which prefer arginine residues or lysine residues; thus detection of its activity with leucine aminopeptidase substrate could be challenging. All in all, the use of different methods, especially substrates and enzyme preparation (extraction, purification) could definitely provide misleading results.

## 6. Conclusions

Plants may directly take up inorganic, but also organic N. Moreover, plants enhance soil proteolysis through root-derived proteases. Future studies should aim to extrapolate laboratory, sterile experiments into heterogenic soil conditions. First attempts suggest that newly depolymerized high-molecular-mass organic N contributes to 20–30% of N uptake by maize, as proven by the addition and uptake of triple labelled (^14^C, ^13^C, ^15^N) high-molecular-mass organic N [71]. Similarly, root-derived proteases may account for up to 20% of the total soil proteolysis [13]. Studies in the future should take into account not only the competition with microorganisms but also the heterogenic soil N sources, including stable soil organic nitrogen pools [72,73], hotspots of proteins, and spatial and temporal variability in N availability due to disturbances. The ability of plants to access organic N sources should be further studied to finally provide effective climate-smart organic nitrogen fertilization recommendations.

## Figures and Tables

**Table 1:** Root-derived enzymes.

Enzyme	Plant Species	Potential Role	References
acid phosphatase	numerous species, for example: *Arachis hypogaea*, *Brasicca oleracea*, *Glycine max*, *Lupinus* sp., *Oryza sativa*, *Pennisetum glaucum*, *Raphanus sativus*, *Sesamum indicum*, *Sinapis alba*, *Solanum lycopersicum*, *Sorghum*, *Triticum aestivum*, *Vigna aconitifolia*, *Vigna radiate*	increase of plant-available P pool (digestion of organic P)	[30,41,42,43,44,45]
phytase	numerous species, for example: *Agrostis gigantea*, *Dactylis glomerata*, *Lupinus albus*, *Medicago sativa*, *Oryza sativa*, *Phleum pretense*, *Solanum lycopersicum*, *Trifolium hybridum*, *Trifolium pratense*, *Trifolium repens*	increase of plant-available P pool (digestion of inositol hexaphosphate)	[46,47]
chitinase glucanase myrosinase	*Arabidopsis thaliana*	defense	[33]
proteases	numerous species, for example: *Allium porrum*, *Allium cepa*, *Zea mays*, *Cucurbita pepo*, *Cucumis sativus*, *Hippopohae rhamnoi-des*, *Geranium pusillum*, *Lactuca sativa*, *Ruta graveolens*, *Raphanus sativus*	increase of plant- available N pool, defense	[33]
root-surface associated protease	*Arabidopsis thaliana*, *Medicago sativa*, *Sinapis alba*	unknown	[34]
	*Triticum eastivum*, *Zea mays*	unknown	[13]
peroxidase, laccase, monophenol mono-oxygenase, superoxide dismutase	*Arabidopsis thaliana*, *Medicago sativa*, *Lepidium sativum*, *Sinapis alba*	oxidative degradation of certain soil components, defense, regulation of allelopathic chemicals	[33,48,49,50]

## Data Availability

Not applicable.
